# Plasma microRNAs as potential biomarkers in early Alzheimer disease expression

**DOI:** 10.1038/s41598-022-19862-6

**Published:** 2022-09-16

**Authors:** Carmen Peña-Bautista, Adrián Tarazona-Sánchez, Aitana Braza-Boils, Angel Balaguer, Laura Ferré-González, Antonio J. Cañada-Martínez, Miguel Baquero, Consuelo Cháfer-Pericás

**Affiliations:** 1 Alzheimer Disease Research Group, Instituto de Investigación Sanitaria La Fe, Avda de Fernando Abril Martorell, 106, 46026 Valencia, Spain; 2 Inherited Cardiomyopathies and Sudden Death Unit, Instituto de Investigación Sanitaria La Fe, Valencia, Spain; 3 Neurology Unit, Hospital Universitario y Politécnico La Fe, Valencia, Spain

**Keywords:** Biochemistry, Biological techniques, Neuroscience, Biomarkers, Neurology

## Abstract

The microRNAs (miRNAs) are potential biomarkers for complex pathologies due to their involvement in the regulation of several pathways. Alzheimer Disease (AD) requires new biomarkers in minimally invasive samples that allow an early diagnosis. The aim of this work is to study miRNAS as potential AD biomarkers and their role in the pathology development. In this study, participants (n = 46) were classified into mild cognitive impairment due to AD (MCI-AD, n = 19), preclinical AD (n = 8) and healthy elderly controls (n = 19), according to CSF biomarkers levels (amyloid β42, total tau, phosphorylated tau) and neuropsychological assessment. Then, plasma miRNAomic expression profiles were analysed by Next Generation Sequencing. Finally, the selected miRNAs were validated by quantitative PCR (q-PCR). A panel of 11 miRNAs was selected from omics expression analysis, and 8 of them were validated by q-PCR. Individually, they did not show statistically significant differences among participant groups. However, a multivariate model including these 8 miRNAs revealed a potential association with AD for three of them. Specifically, relatively lower expression levels of miR-92a-3p and miR-486-5p are observed in AD patients, and relatively higher levels of miR-29a-3p are observed in AD patients. These biomarkers could be involved in the regulation of pathways such as synaptic transmission, structural functions, cell signalling and metabolism or transcription regulation. Some plasma miRNAs (miRNA-92a-3p, miRNA-486-5p, miRNA-29a-3p) are slightly dysregulated in AD, being potential biomarkers of the pathology. However, more studies with a large sample size should be carried out to verify these results, as well as to further investigate the mechanisms of action of these miRNAs.

## Introduction

MicroRNAs (miRNAs) have been postulated in recent years as good biomarkers for the diagnosis, prognosis and therapies of different pathologies^1^. They are non-coding 19–25 nucleotides RNA molecules, which are involved in the regulation of gene expression^2^.

Alzheimer’s disease (AD) is the most common dementia in developed countries, being one of the leading causes of death, disability and dependency in older populations^3^. However, despite the efforts and economic investments made in research into this pathology, it is a complex pathology in which several factors are involved and whose mechanisms are not fully understood^4^. Specifically, the most consolidated mechanisms are those involved in the accumulation of amyloid-β42 peptide and phosphorylated Tau (p-Tau) in brain^5^. Nevertheless, other mechanisms such as oxidative stress, neuroinflammation or lipid metabolism could contribute to the pathology^5–8^. Nowadays, the diagnosis of AD is complex and relies on cerebrospinal fluid (CSF) biomarkers (amyloid-β42, t-Tau, p-Tau) levels, neuroimaging (NMR, PET), and neuropsychological assessment^9–12^. Thus, further research focused on minimally invasive biomarkers is required^13^.

Regarding physiopathological mechanisms involved in AD development, miRNAs have been postulated as mediators. Each miRNA could be involved in different pathways as it can have different target genes. In fact, a miRNA could recognize a regulatory region in different genes, regulating, activating or inactivating their expression. Specifically, some miRNAs have been related to the regulation of amyloid protein precursor (APP) cleavage, presenilin-1 (PSEN1) and beta-site amyloid precursor protein cleaving enzyme 1 (BACE1), as well as in oxidative stress and other AD risk factors^14^. In addition, differential expression of miRNAs in AD could be useful in the diagnosis of the pathology, and some miRNAs have been described as potential biomarkers for AD diagnosis and prognosis^15^. Some miRNAs showed good performance as biomarkers, mainly panels including different miRNAs might be dysregulated several years before the onset of disease symptoms^16^. Several panels were developed from plasma, serum or exosomes, showing their potential for a minimally invasive disease diagnosis^17–19^. In general, different results have been found in the literature, probably due to the lack of diagnostic biomarkers used in the classification of participants or due to the differential methodologies applied in sequencing or data processing.

The aim of this work is to analyse the differential expression of a panel of miRNAs selected from sequencing analysis in plasma from AD and non-AD patients, evaluating their potential usefulness as biomarkers or in the development of therapeutic targets, as well as to study their potential implications in the course of the AD.

## Material And Methods

### Participants and samples collection

In this study, participants (n = 46) were aged between 50 and 80 years old and were classified into mild cognitive impairment due to AD (MCI-AD, n = 19), preclinical-AD (n = 8) and healthy controls (n = 19), according to the National Institute on Aging and Alzheimer's Association (NIA-AA) criteria. Briefly, the control group showed negative CSF biomarkers or amyloid PET, and normal neuropsychological assessment; the MCI-AD group showed positive CSF biomarkers or amyloid PET, and impaired neuropsychological assessment; the preclinical-AD group showed positive CSF biomarkers or amyloid PET, and normal neuropsychological assessment. Participants with other major disorders and those unable to complete the assessment were excluded. All participants were recruited at the Neurology Unit of the University and Polytechnic Hospital La Fe (Valencia, Spain) after signing informed consent, and plasma samples were obtained at the same time the lumbar puncture. The study protocol (2019/0105) was approved by the Ethics Committee (CEIC) of the Instituto de Investigación Sanitaria La Fe (Valencia, Spain). No randomisation was performed to allocate subjects in the study. No pre-registration was performed. The study is blinded as the classification of participants was performed by a person other than the experimenter. No sample calculation was performed. All methods were carried out in accordance with relevant guidelines and regulations.

### RNA extraction

RNA was isolated for RNA sequencing using the miRNeasy plasma kit (Qiagen, Germany) following the manufacturer’s protocol. Briefly, 200 µL of plasma and 700 µL of QIAzol lysis reagent were incubated for 5 min at room temperature (RT). Then, 140 µL of chloroform were added and incubated at RT for 3 min and centrifuged at 1200 g (15 min, 4 °C). The aqueous phase was mixed in a new tube with 525 µL of ethanol and transferred to a RNeasy MinElute spin column followed by a centrifugation step at 10000 g (30 s, RT). The column was then washed with RWT buffer (700 µL) and RPE buffer (500 µL) and dried for 90 s at 10000 g. Finally, the elution step was performed with 15 µL of RNase-free water (13000 g, 1 min).

For PCR validation, RNA extraction was carried out in a similar way but including a previous step, which consisted on the addition of RNS spike-in before the protocol.

### RNA sequencing method

#### Construction of RNA libraries

The miRNA libraries were prepared from total RNA using the NEXTFLEX® Small RNA-Seq v3 Kit for Illumina Platforms (Bioo Scientific Corporation, Texas, USA). Briefly, the small RNA molecules were first ligated to the 3'-4 N adenylated adapters, taking advantage of the phosphate group at their terminal end, which allows the exclusive targeting of these molecules. Secondly, the 5'-4 N adapters were ligated. Later, reverse transcription of the molecule into cDNA was carried out. The generated cDNA fragments were then amplified and indexed by PCR using different barcode primers for each sample. Finally, a size-selective purification was carried out.

The quality control and concentration of the libraries were verified with the Agilent Technologies 2100 bioanalyser using highly sensitivity DNA chips (Central Unit for Research in Medicine (Universitat de València)). Subsequently, an equimolecular pool of each library was prepared for sequencing.

#### Sequencing on an Illumina equipment

Sequencing was carried out on the NGS NextSeq 550 platform (Illumina, San Diego, CA, USA) by single read sequencing of 50 cycles (1 × 50 bp).

### Data analysis

#### Pre-processing, quality control and normalization

NGS data (raw fastq files from sRNA sequencing) were processed following the standard protocol proposed by Cordero et al*.*^20^ implemented in the function mirnaCounts from docker4seq package^21^ with default parameters in R^22^. First, a sequence quality control check was generated using FastQC^23^ and then cutadapt^24^ program was used for the adapter trimming. Specifically, adapters and low-quality reads (Phred Score < 10) were trimmed and removed (44.014.980 reads). Once adapters were removed, sequence reads (219.207.246 good quality reads) were mapped against miRNA precursors from miRBase (v.21)^25^, using SHRIMP^26,27^, filtering out a total of 95,03% reads. Finally, miRNA quantification from the resulting 4,97% of mapped reads were generated using the function count Overleaps from GenomicRanges package^28^, resulting in a total of 9.799.858 miRNA counts in a total of 2.386 miRNAs.

#### miRNAs selection

From the miRNAs identified in the pre-processing, quality control and normalization process, some of them were selected. Specifically, those miRNAs which showed a number of counts different from zero in at least 80% of the samples and that were corroborated in literature. Finally, the selected miRNAs were validated by means of qPCR in the same plasma samples.

### miRNAs validation by quantitative PCR

#### Quantitative PCR procedure

From the extracted RNA, retrotranscription and amplification steps were carried out following the manufacturer’s recommendation (TaqMan Advanced miRNA Assays) [https://tools.thermofisher.com/content/sfs/manuals/100027897_TaqManAdv_miRNA_Assays_UG.pdf]. Briefly, the protocol consisted of four steps. First, the addition of a polyA tail, after the adapter ligation, followed by the retrotranscription step, and then the specific miRNA amplification. Finally, samples were diluted, and real time PCR (RT-PCR) was carried out in duplicate using the thermocycler (ViiA7, Applied Biosystems, California, USA).

#### Statistical analysis

The number of counts obtained from RT-PCR were averaged for duplicates, discarding replicates with values within ± 2 counts from mean. Then, samples were normalized using the mean and standard deviation. The miRNAs detected in at least 80% of the samples and with a difference between replicates < 1 count were considered satisfactorily quantified. The effect of each biomarker on pathology was then analyzed by Bayesian models: the first model discriminates among control, MCI-AD and preclinical AD groups; and the second model discriminates between AD (preclinical AD, MCI-AD) and control groups. For these models, some parameters were calculated (estimate, which indicates the direction of the miRNAs levels; Odds Ratio; Percentage Inside Rope, which defines the percentage of the area that is within the region of practical equivalence (equivalent to null effect); probability of direction (PD), which indicates the probability that the effect has in a particular direction (indicated by the estimate). PD > 80% was considered significative).

#### Pathway analysis

The target genes of the differentially expressed miRNAs were studied using the miR data base (miRDB). The selected target genes were those with a target score ≥ 95. Then, the targets were classified according to cellular pathways and functions in order to analyze the implication in AD.

### Ethics approval

The study protocol (2019/0105) was approved by the Ethics Committee (CEIC) from Health Research Institute La Fe (Valencia, Spain).

### Consent to participate

Informed consent was obtained from all individual participants included in the study.

### Research involving Human Participants and/or Animals

Yes, human participants.

### Informed consent

All the participants were recruited in the Neurology Unit from University and Polytechnic Hospital La Fe (Valencia, Spain) after signing the informed consent.

## Results

### Participants characteristics

The participants’ characteristics are summarized in Table 1. As can be seen, most of the variables showed no significant differences among participants’ groups. In fact, only the clinical variables used in their diagnosis (CSF biomarkers levels, neuropsychological assessment) show statistically significant differences, as expected. In contrast, demographic variables (age, sex, educational level, medication use (statins, fibrates, benzodiazepines, antihypertensives), comorbidities (dyslipidemia, diabetes, hypertension)) are similar between the study groups.

**Table 1:** Participant’s clinical and demographic variables.

Variable	Control (n = 19)	MCI-AD (n = 19)	Preclinical-	*P* value
	Median (1st, 3rd Q.)			
Age (years)	69 (64.5, 70.5)	70 (67.5, 74)	68.5 (66.7, 70.5)	0.134
Sex, female, n (%)	8 (42.11%)	8 (42.11%)	5 (62.5%)	0.575
**Educational level (n, %)**				
Basic or primary	6 (31.58%)	7 (38.89%)	1 (12.5%)	0.094
Secondary	6 (31.58%)	10 (55.56%)	3 (37.5%)	
Uiversitary	7 (36.84%)	1 (5.56%)	4 (50%)	
Smoking Yes, n, (%)	3 (15.79%)	3 (15.79%)	2 (25%)	0.823
Alcohol Yes, n (%)	4 (21.05%)	2 (10.53%)	1 (12.5%)	0.647
Statins (n, %)	11 (57.89%)	10 (52.63%)	3 (37.5%)	0.625
Fibrates (n, %)	2 (10.53%)	2 (11.11%)	1 (14.29%)	0.690
Benzodiazepines (n, %)	3 (15.79%)	2 (10.53%)	1 (12.5%)	0.889
Antihipertensives (n, %)	8 (42.11%)	7 (38.89%)	1 (12.5%)	0.317
Dyslipidemia (n, %)	13 (68.42%)	10 (52.63%)	3 (37.5%)	0.303
Diabetes (n, %)	3 (15.79%)	1 (5.26%)	3 (37.5%)	0.103
Hypertenison (n, %)	9 (47.37%)	8 (42.11%)	1 (12.5%)	0.224
Amyloid-β42 (pg mol-1)	1224 (967, 1429)	495 (456, 616)	671.5 (507.5, 714)	< 0.001
t-Tau (pg mol-1)	276 (227.5, 375)	578 (432.75, 785.75)	464 (337.5, 548.5)	0.001
p-Tau (pg mol-1)	40 (29, 44)	91 (58.75, 107.75)	67 (58.25, 99)	< 0.001
CDR	0 (0, 0)	0.5 (0.5, 0.5)	0 (0, 0)	< 0.001
MMSE	29 (27.5, 29.5)	24 (23, 25.75)	27 (26.75, 28.25)	< 0.001
FAQ	0 (0, 1)	7 (5, 10.5)	1 (0, 2)	< 0.001
RBANS.MR	101 (96.5, 106.5)	42 (40, 55)	86 (77.25, 98.75)	< 0.001

### miRNAs validation

A panel of 11miRNAs was selected following the specified criteria (counts in at least 80% of the samples and previous findings in literature). The selected miRNAs were hsa-miR-92a-3p, hsa-miR-486-5p, hsa-miR-29a-3p, hsa-miR-486-3p, hsa-miR-150-5p, hsa-miR-142-5p, hsa-miR-320b, hsa-miR-483-3p, hsa-miR-1293, hsa-miR-342-3p, and hsa-miR-4259. Of these, 8 miRNAs were successfully quantified (has-miR-92a-3p, has-miR-486-5p, has-miR-29a-3p, miR-486-3p, miR-150-5p, miR-320b, miR-483-3p, miR-342-3p); while some miRNAs were not detected (hsa-miR-142-5p, miR-1293, hsa-miR-4259). The levels obtained for each miRNA are summarised in Table 2. As can be seen, small differences were obtained for each miRNA among participants’ groups.

**Table 2:** Median levels of miRNAs in plasma from participants’ groups.

Variable (Total counts)	Control (n = 19)	MCI-AD (n = 19)	Preclinical AD (n = 8)
	Median (1st, 3rd Q.)		
hsa-miR-92a-3p	22.26 (21.12, 22.67)	21.51 (21.27, 22.72)	21.89 (21.37, 22.61)
hsa-miR-486-5p	22.72 (22.22, 23.43)	22.5 (22.13, 23.3)	23.33 (22.26, 24.21)
hsa-miR-29a-3p	26.86 (25.92, 27.55)	26.93 (26.4, 27.36)	27.62 (26.62, 27.99)
hsa-miR-486-3p	28.19 (27.47, 28.96)	28.07 (27.44, 29.35)	27.98 (27.4, 29.8)
hsa-miR-150-5p	24.18 (23.84, 24.9)	23.93 (23.38, 25.2)	23.93 (23.38, 24.49)
hsa-miR-320b	26.94 (26.26, 27.64)	26.73 (26.19, 27.1)	26.88 (25.94, 27.48)
hsa-miR-483-3p	31.53 (31.18, 32.32)	31.63 (30.97, 32.91)	31.5 (31.31, 31.74)
hsa-miR-342-3p	28.54 (28.07, 29.04)	28.48 (27.7, 29.46)	27.71 (27.05, 28.75)

Individually, the validated miRNAs showed no significant differences between groups. Therefore, two multivariate models, including the previously selected miRNAs, were developed to analyse the tendency of each miRNA in participants’ groups. The first model included 3 participant groups (control, MCI-AD, preclinical AD); while the second model included 2 participant groups (AD (MCI-AD + preclinical-AD), control). In Table 3, the characteristics of the first model are summarised, showing that the miRNAS hsa-miR-92a-3p, hsa-miR-486-5p and hsa-miR-29a-3p had a high probability of direction (PD > 80%). Specifically, hsa-miR-92a-3p showed a PD 85.40% of a negative estimate, so relatively reduced levels were found in AD. Similar results were obtained for hsa-miR-486-5p. In fact, it showed a high probability of a negative estimate with small Region of Practical Equivalence (ROPE) (< 15%), which defines the percentage of the area that is within the region of practical equivalence (equivalent to null effect)), showing an Odds Ratio (OR) lower than 1, and suggesting a protective effect for AD. By contrast, hsa-miR-29a-3p showed a positive estimate, so relatively increased levels were found in AD. Similarly, the characteristics of the model including 2 participants’ groups (AD, control), showed that the miRNAS hsa-miR-92a-3p and hsa-miR-29a-3p had a PD > 90%, with negative and positive estimates, respectively.

**Table 3:** Characteristics of the Bayesian model including 3 participants groups (control, preclinical-AD, MCI-AD).

Variables	Estimate	OR (CI 95%)	Inside Rope (%)	PD (%)
hsa-miR-92a-3p	−0.484	0.616 (0.241,1.455)	19.34%	85.40%
hsa-miR-486-5p	−0.649	0.522 (0.112,2.28)	14.15%	81.38%
hsa-miR-29a-3p	0.418	1.519 (0.662,3.626)	22.76%	82.88%
hsa-miR-486-3p	0.478	1.613 (0.462,5.929)	18.05%	77.88%
hsa-miR-150-5p	0.123	1.131 (0.243,5.574)	19.76%	55.27%
hsa-miR-320b	0.174	1.19 (0.373,4.02)	23.34%	60.68%
hsa-miR-483-3p	0.286	1.331 (0.624,2.968)	29.86%	77.15%
hsa-miR-342-3p	−0.458	0.632 (0.131,3.086)	16.47%	72.58%

The Probability of Direction (PD) is an index of effect existence, ranging from 50 to 100%, representing the certainty with which an effect goes in a particular direction. PD > 80% was considered significative. For each variable the direction depends on the estimate (negatives estimate < 0, and positives estimates > 0). Region of Practical Equivalence (ROPE) defines the percentage of the area that is within the region of practical equivalence (equivalent to null effect).

*OR *odds ratio, *CI* confidence interval.

**Figure 1:**
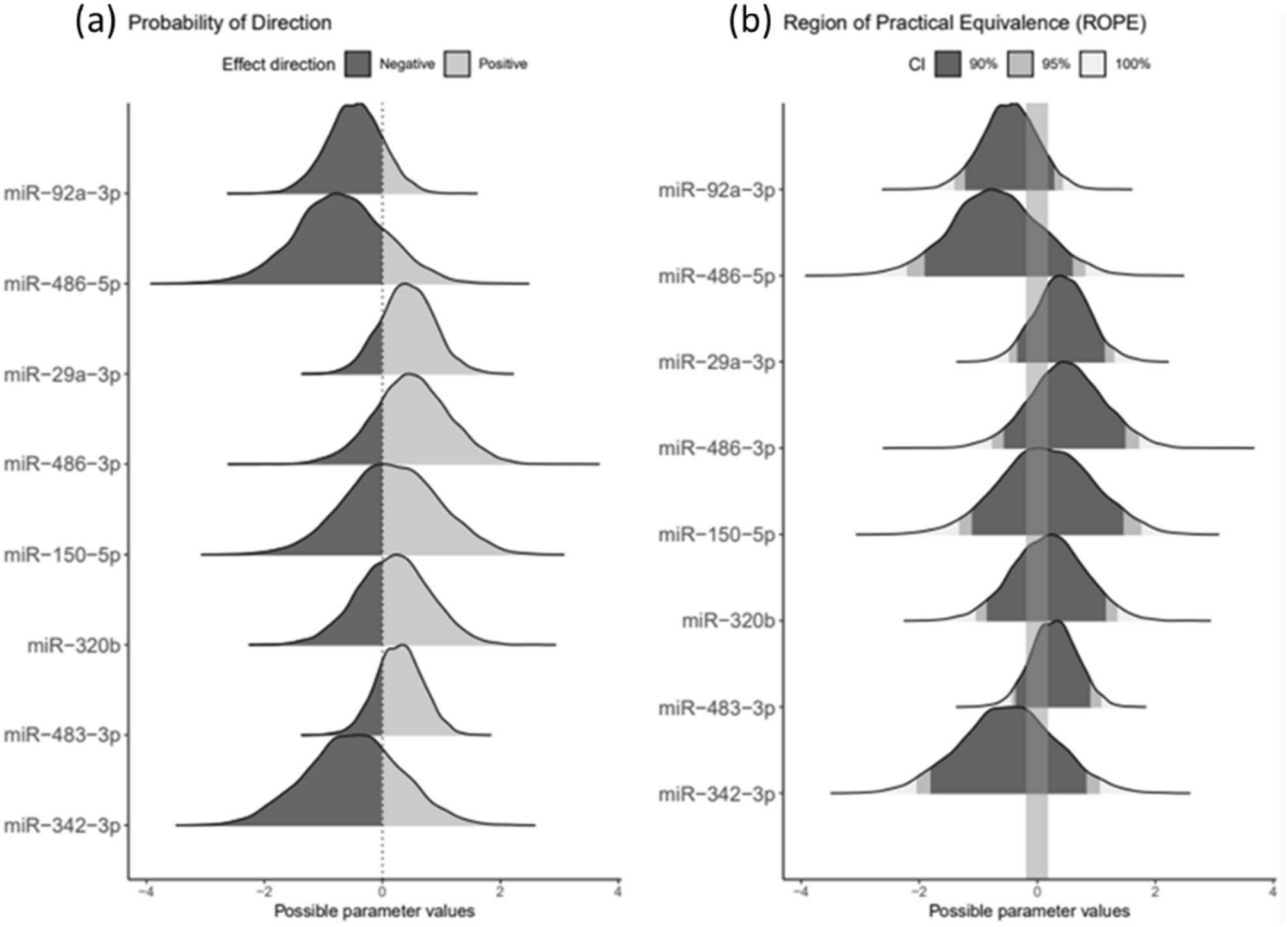
Probability of direction (PD) and Region of Practical Equivalence (ROPE) for each miRNA. (**a**) PD shows the estimation of direction for each biomarker, showing a protective AD effect for those with negative direction and risk AD effect for those with positive direction. Polygons show the density summary of the posterior draws and coloured given the estimated direction (positive or negative) of the effect parameter. The proportion of the polygon that does not include zero is a statement about probability of the proposed direction of effect. (**b**) ROPE represents the area of null equivalence that is the percentage with none direction (positive or negative). Effects given a full ROPE based on a 100%, 95% and 90% highest posterior density interval. The proportion of the polygon that does not include zero is a statement about the significance of effect.

These results are shown in Fig. 1, which depicts the PD and ROPE for each miRNA. The miRNAs with a high PD (mir-92a-3p, miR-486-5p, miR-29a-3p), showed most of their area on one side of 0 (Fig. 1a). In addition, mir-92a-3p and miR-486-5p showed a negative direction, while miR-29a-3p showed a positive direction. Figure 1b shows the ROPE region, being a small area in the first three miRNAs.

### Pathway analysis

**Figure 2:**
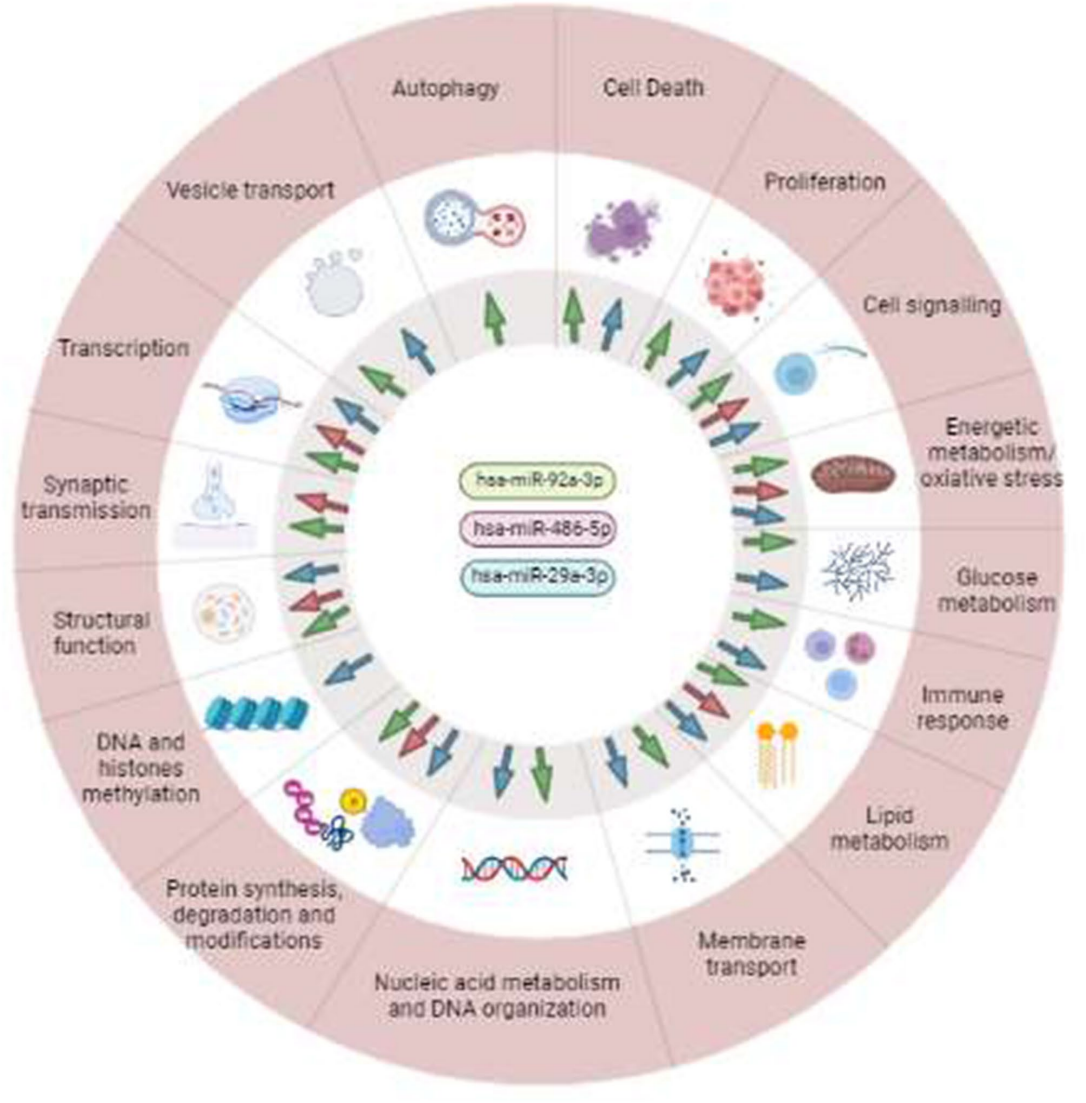
Pathways regulated by the three miRNAs that showed relationship with AD. The arrows indicate those miRNAs involved in each pathway. Each color represents a miRNA: green (hsa-miR-92a-3p), red (hsa-miR-486-5p) and blue (hsa-miR-29a-3p). *Created with BioRender.com.

For the miRNAs with a high directional probability (has-92a-3p, has-486-5p, has-29a-3p), their potential target genes were analysed in order to assess their involvement in the pathology development. Table 4 shows the potential target genes of the selected miRNAs related to AD mechanisms. As can be seen, 112 potential targets were obtained for miRNA hsa-92a-3p, 16 targets for hsa-486-5p, and 88 targets for hsa-29a-3p, with a target score of at least 95. In addition, each of the selected miRNAs regulated several pathways. As can be seen in Fig. 2, the most common pathways were cell signalling and transcription regulation, but also lipid metabolism, protein synthesis and modifications, and structural functions were regulated by the selected miRNAs. First, the main pathways that could be regulated by the miRNA hsa-92a-3p are cell death or autophagy and cell proliferation pathways, and some pathways related to vesicle transport and synaptic transmission. Among the cell death targets, BCL2L11 (BCL2 like 11) is involved in neuronal and lymphocyte apoptosis and G3BP2 (G3BP stress granule assembly factor 2) is involved in stress response. In the cell proliferation pathway, the gene C21orf91 (chromosome 21 open reading frame 91) plays a role in the proliferation of neurons in the cortex. Among synaptic transmission targets, GLRA1 (glycine receptor alpha 1), SYN2 (synapsin II), SCN8A (sodium voltage-gated channel alpha subunit 8), CADM2 (cell adhesion molecule 2), CBLN4 (cerebellin 4 precursor), SYNJ1 (synaptojanin 1), SLC17A6 (solute carrier family 17 member 6), and NSF (N-ethylmaleimide sensitive factor, vesicle fusing ATPase) are highlighted, being the last two targets involved in vesicle transport. Other important genes are REST (RE1 silencing transcription factor), which regulates neuronal genes transcription; and NEFH (neurofilament heavy), which contributes to the maintenance of neuronal structure. In addition, PPCS (phosphopantothenoylcysteine synthetase) could be relevant in the regulation and metabolism of CoenzymeA.

**Table 4:** Potential target genes and related AD pathways.

Pathway	hsa-miR-92a-3p	hsa-miR-486-5p	hsa-miR-29a-3p
Autophagy	TECPR2, EPG5		
Cell death	G3BP2, HIPK3, USP28, DNAJB9, BCL2L11, RNF38		TRIB2, XKR6, AKT3
proliferation	CD69, FNIP1, BTG2, MAP2K4, C21orf91, KLF4, FNIP2, GTF2A1, CDK16, ARID1B, CDCA7L, CCNJL, CUX1, MAP1B, RNF38		NAV1, NAV2, NAV3, IGF1, ZNF346, LIF, CDK6, SGMS2, PDIK1L, CHSY1, NEXMIF, AKT3, ADAMTS9
Cell signalling	PIKFYVE, DOCK9, ITGAV, EFR3A, RIC1, RNF38, GPR180, PLEKHA1, JMY, GNAQ, RGS17, PTEN, PCDH11X, GIT2, ADGRF2, CALN1, DPP10, LRCH1, HCN2	DCC, PTEN, SLC10A7, ARHGAP44, MARK1	NEXMIF, AKT3, DAAM2, PTEN, PGAP2, ROBO1, RAP1GDS1, RAB30, DGKH, CLDN1, TRAF3
Energetic metabolism and oxidative stress	NOX4, SESN3, PTEN, SLC12A5	PTEN	PTEN
Glucose metabolism	MAN2A1, FBN1, UGP2		FBN1
Immune response	TAGAP, CD69, KLF4, GLRA1, FOXN2, RAB23		TRAF3
lipid metabolism	PPCS, KIAA1109	FAHD1	OSBPL11
membrane transport	SLC12A5, SLC25A32, SGK3		SESTD1, ABCE1, SLC5A8
Nucleic acid metabolism and DNA organization	MORC3, RBM27, GID4, CPEB3, SLX4, AGO3, JMY, ANP32E, RSBN1		DOT1L, KMT5C, ERCC6, NASP, KDM5B, TDG
DNA and histones methylation			TET1. TET2, TET3, DOT1L, DNMT3A, DNMT3B, KDM5B
Protein degradation	FBXW7, SESN3, KLHL14, USP36, USP28, UBXN4		VPS37C, TRIM63
Protein synthesis and modifications			
	B3GALT2, PTAR1, GOLGA3, COG3, SGK3, ADAM10, EDEM1	COPS7B, MARK1, LMTK2, ABHD17B	ADAMTS9, ADAMTS6, DIO2, ABCE1
Structural function	ACTC1, ANP32E, NEFH, RSBN1, NCKAP5, NEFM, RHPN2, FBN1, MYO1B	SNRPD1, NCKAP5, LCE3E	COL5A3, COL5A1, COL3A1, FBN1, COL11A1, HAS3, TMEM169, COL19A1, COL4A1, COL1A1, COL7A1, SPARC, COL5A2, HMCN1, C1QTNF6, ADAMTS2, CEP68, PXDN, COL9A1, HAPLN3, RND3, TRAF3, RAB30, CLDN1
Synaptic transmission	GLRA1, SYN2, SCN8A, CADM2, CBLN4, SYNJ1, SLC17A6, NSF	ARHGAP44	
Transcription	MIER1, HAND2, TBL1XR1, LATS2, FOXN2, ZEB2, REST, GRHL1, TEAD1, HIVEP1	BTAF1, SNRPD1, FOXO1, ZNF331	HBP1, ATAD2B, BRWD3, NSD1, ZBTB34, NFIA, KDM5B, PURG, HIF3A, ZBTB5, ZNF282, AMER1, REST, TAF5, ZHX3, C16orf72
Vesicle transport	MYO1B, CDK16, PIKFYVE, SLC17A6, NSF, RAB23, DENND1B		ASAP2, VPS37C
Others	ZFC3H1, TTC9, ATXN1, DCAF6, LHFPL2, FAM160B1, ERGIC2, MAGEC2, SPRYD4, ANKRD28, TRIM36, FAM24A, BCL11B	TRIM36	ADAMTS17, PRR14L, FAM241A, LYSMD1, PXYLP1, SMS, ATAD2B

In this link it can be found the full name of each gene (http://mirdb.org/mirdb/index.html).

Secondly, the main pathways that could be regulated by the miRNA hsa-486-5p are cell signalling, lipid and protein pathways, structural functions and transcription.

Thirdly, the main pathway that could be regulated by the miRNA hsa-29a-3p is the cell proliferation pathway, which involves neurone regeneration and migration trough NAV3 (neuron navigator3), NAV1, and NAV2. Also, ZNF346 (zinc finger protein 346) could act to protect neurons and LIF (LIF, interleukin 6 family cytokine) is involved in neuronal differentiation. In cell signalling pathways, the targets DAAM2 (dishevelled associated activator of morphogenesis 2) and ROBO1 (roundabout guidance receptor 1) contribute to nervous system development and neuronal migration, respectively. Furthermore, miRNA hsa-29a-3p plays a role in structure regulation, specifically regulating the synthesis of different collagen chains, and HMCN1 (hemicentin 1) is involved in macular degeneration and C1QTNF6 (C1q and TNF related 6) is involved in identical protein binding activity. Also, this miRNA could regulate REST in the transcription pathway.

## Discussion

In this study, miRNA sequencing was carried out to identify potential early AD biomarkers. From these, a validation step was conducted, in which quantifiable miRNAs were identified, while some of them were not detected. In fact, the miRNAs not validated were hsa-miR-142-5p, hsa-miR-1293 and hsa-miR-4259. A previous study in cell line found a relationship between dysregulation of miR-142-5p expression and AD pathogenesis and synaptic dysfunction^29^, and it was detected up-regulated in the blood of AD patients^30^. Also, hsa-miR-4259 was detected in saliva samples, but there is a lack of studies quantifying this biomarker in plasma samples^31^. In addition, has-miR-1293 was previously detected in platelets from hepatocellular carcinoma and lung adenocarcinoma cell line^32^. Nevertheless, there are no studies describing its association with AD.

Regarding the methodology, Haining et al., performed a similar study trying to find a miRNA profile in early AD. However, different cohorts for untargeted and targeted analysis were used^17^. Also, Dakterzada aimed to find miRNAs in plasma from AD participants, identifying a BACE-1 related panel of biomarkers different from the miRNAs in the present work^33^. It could be due to the use of a different identification technique based on microarrays analysis^34^. The different methodologies employed could affect the miRNAs selection, so it should be taken into account in comparisons with other studies^33^.

Regarding the miRNAs that showed a trend with the pathology in the present study, they were hsa-miR-92a-3p, has-miR-486-5p and hsa-miR-29a. First, hsa-miR-92a-3p showed a tendency for decreased levels in AD. A previous study showed dysregulation of 3 miRNAs related to synaptic proteins, including hsa-miR-92a-3p in MCI and AD^35^. Another study described the relationship between miR-92a-3p and tau accumulation^36^. One of the most AD-relevant pathways that could be regulated by this miRNA is synaptic transmission^37^. Specifically, SYNJ1, a potential target for this miRNA, seemed to be involved in amyloid beta clearance^38,39^, while synapsins could act on amyloid beta generation by modulating BACE1^40^. In addition, CBLN4 could regulate amyloid beta toxicity^41^. Regarding neuronal apoptosis, it could be regulated by this miRNA and the BCL2L2 target. In fact, a previous work showed that amyloid beta could regulate that pathway^42^. Other target genes (NEFH, REST), which are involved in neuronal structure and neuronal gene transcription, were described as potential AD diagnosis biomarkers^43,44^.

Second, the present study showed a tendency towards reduced levels of hsa-miR-486-5p in AD. Similarly, Nagaraj et al. described a panel of 6 plasma miRNAs, including hsa-miR-486-5p, that differentiated between controls and MCI-AD^45^. This miRNA could regulate some genes involved in cell signalling, lipid and protein pathways, transcription and structural function.

Third, a trend towards higher levels for hsa-miR-29a-3p in AD plasma was found. Similarly, Shioya et al. described differential levels of this miRNA at brain levels, suggesting its implication in neurodegeneration trough NAV3 (Neurone Navigator 3) regulation^46^. In addition, another miRNA from that family (hsa-miR-29c) has been related to AD pathology due to its involvement in the amyloid beta accumulation through the regulation of BACE1^47,48^. Moreover, Müller et al. suggested that miR-29a could be a candidate biomarker for AD in CSF samples without cells^49^. In this regard, different types of collagenous chains and C1QTNF6 are targets of miRNA hsa-29a-3p. Previous studies described collagenous chains as a component from amyloid plaques^50^. The collagenous regulation may contribute to the assembly of amyloid fibres, enhancing the development of amyloid pathology. In addition, C1q complement protein co-localizes with the amyloid beta in brain^51,52^. Therefore, C1QTNF6, which is thought to play a role in identical protein binding, could help in the accumulation of C1q protein, triggering amyloid plaque formation (PubMed Gene). In addition, ROBO1 and DAAM2, which are involved in neuronal migration and nervous system development, are targets for this miRNA. In fact, ROBO1 could show a relationship with axon guidance dependent on presenilin, which helps in the proteolysis of amyloid-β precursor protein and triggers to AD pathology development^53,54^. Furthermore, DAAM2 was described by Ding et al. as a mediator in regenerative oligodendrocyte differentiation; while Sellers et al. demonstrated that Aβ synaptotoxicity is mediated by this protein^55,56^.

The main limitations in this study are the small sample size, since it is quite difficult to have a large number of biologically classified early AD patients (MCI, preclinical). Moreover, from the selected miRNAs, some of them were not validated as they were not correctly quantified, probably due to the fact that they were detected in few samples. In addition, the study design is cross-sectional. In order to obtain more accurate data from the different disease stages, it should be longitudinal. However, participants in this present study are perfectly characterized according to CSF biomarkers and their cognitive status, providing a reliable approach to the disease progression.

## Conclusions

RNA sequencing analysis in plasma samples from participants with early AD and healthy controls allowed to identify some differentially expressed miRNAs. From them, 3 selected miRNAs (miRNA-92a-3p, miRNA-486-5p, miRNA-29a-3p) were slightly dysregulated in AD, being potential biomarkers of the pathology. In fact, they could be involved in the regulations of important pathways of the pathology, such as synaptic transmission, cell signalling, structure maintenance or cell metabolism, so they could be relevant therapeutic targets. However, further research with a larger sample is needed to verify these results, as well as to develop the potential mechanisms of action of these miRNAs.

## Data Availability

The datasets generated during the current study are available in the ArrayExpress repository, accession number E-MTAB-11103.
